# Circularly-polarized 28-GHz antenna for next generations of communication systems

**DOI:** 10.1038/s41598-025-86236-z

**Published:** 2025-02-17

**Authors:** Rania R. Elsharkawy, Khalid. F. A. Hussein, Asmaa E. Farahat

**Affiliations:** 1 Microstrip Circuits Department, Electronics Research Institute (ERI), Cairo, Egypt; 2 Microwave Engineering Department, Electronics Research Institute (ERI), Cairo, Egypt

**Keywords:** Engineering, Physics

## Abstract

A circularly-polarized (CP) printed antenna is proposed for millimeter-wave mobile communications. The antenna has a defected ground structure (DGS), and it operates at 28 GHz. The geometries of the antenna on the upper surface and the DGS are symmetric about a $45^\circ$-diagonal to produce perfect circular polarization. The antenna is fed through a three-stage microstrip line that acts as an impedance transformer for perfect matching of the antenna impedance to $50\,{\Omega\:}$ at $28\:\text{G}\text{H}\text{z}$. The development of the antenna design is described in detail, starting with a square patch that is subjected to progressive geometrical modifications in the design stages to reach the final design of the patch antenna. The simulation results show that the antenna has a reflection coefficient magnitude of less than $-35\:\text{d}\text{B}$ at an axial ratio of less than 0.5 dB at 28 GHz. The technique used to generate the radiated circular polarized waves is demonstrated by investigating the surface current on the printed patch at the resonant frequency. The primary objective of the proposed design is not to produce a high-gain antenna, but rather to produce a low-profile planar antenna to serve as a building block for CP antenna systems such as multiple-input-multiple-output (MIMO) antennas, beam steering, and beamforming antenna arrays operating at 28 GHz. The designed antenna is fabricated and subjected to experimental evaluation. The numerical simulation results and the practical measurements came in good consent from the impedance matching and axial ratio (AR) of the radiated circular polarized wave perspectives. The input impedance is matched to the feeding line over a 1-GHz band, from 27.5 GHz to 28.5 GHz. The radiated wave is circularly polarized with AR less than 3 dB over about 0.4 GHz band starting from 27.8 GHz to 28.2 GHz. The peak gain of the designed antenna is 7 dBi and the radiation efficiency exceeds 90% all over the band of impedance matching. The performance of the antenna is compared with those of other antenna designs presented in some recent publications.

## Introduction

The demand for millimeter-wave (mm-wave) frequencies in wireless communications is continuously growing at a high rate to enable transfer of large-size data at high speed. The frequency band around 28 GHz is the most important among the other mm-wave frequencies, as it has attracted most of the research efforts and scientific investigations in wireless communication applications starting from the fifth generation (5G) and beyond. Practical experiments have proven that the frequency band around 28 GHz is suitable for mm-wave applications in cellular communication systems^1,2^. In this regard, and specifically in 2006, the Federal Communication Community (FCC) allocated the frequency band from 27.5 to 28.35 GHz for 5G applications in wireless communication systems. The importance of this frequency band comes from its distinctions as follows. Firstly, it has spectral availability, as several sub-frequency bands within this band, which include tens or hundreds of megahertz, can be used for different future applications. Secondly,  since the frequencies 26 and 28 MHz are the lowest mm-wave frequencies available, the problems of wave propagation in the atmosphere are least at these two frequencies, when compared to the higher mm-wave frequencies. In addition, as these two frequencies are the lowest among the other mm-wave frequencies, they are utilized in communication devices with less complexity than those of the devices used at higher mm-wave frequencies^1,3^.

Many applications in wireless communications require circular polarization of the transmitting and receiving antennas, especially when the propagating wave passes through ionized media or charged particles causing depolarization (Faraday rotation). Also, circular polarization is preferred, when it is difficult to align the transmitting and receiving antennas, which would result in severe polarization loss if linear polarization was utilized. In such situations, the utilization of circular polarization enhances the communication system performance.

A lot of the recently-published research work is interested in the issue of design and investigation of CP antennas working at $28\:\text{G}\text{H}\text{z}$. For example, the work of^4^ presents an omnidirectional mm-wave antenna to operate for 5G device-to-device wireless communications. The antenna produces right-hand circular polarization (RHCP) at $28\:\text{G}\text{H}\text{z}$. The omnidirctionality and circular polarization are performed by integrating symmetric electric and magnetic dipoles into a low-profile disc-shaped radiating structure. In^5^, a dielectric resonator antenna (DRA) on a partial ground plane is presented to produce circular polarization over the frequency band of $27.95-28.37\:\text{G}\text{H}\text{z}$ with 97% radiation efficiency. The work of^6^ introduces a square patch antenna to provide circular polarization over the frequency band of $27.0-28.8\:\text{G}\text{H}\text{z}$. In this design, a quarter-wavelength impedance transformer is used for impedance matching over the frequency band of operation. In^7^, a circular patch antenna of modified shape on a DGS is presented to provide circular polarization over the frequency band of $27.8-28.2\:\text{G}\text{H}\text{z}$ with 91% radiation efficiency. The $45^\circ$-diagonal symmetry of the patch geometry and the DGS lead to exciting two simultaneous resonant modes with fields of orthogonal directions, equal magnitudes and $90^\circ$ phase shift, thereby producing circular polarization at 28 GHz. In this design, a tapered microstrip line feeder is utilized to achieve impedance matching over the operational frequency band.

The work in this paper is concerned with the design and implementation of a compact CP antenna operating at $28\:\text{G}\text{H}\text{z}$. The proposed antenna is a patch antenna printed on a thin substrate with DGS. The geometries of the antenna on the upper surface and the DGS are symmetrical about a diagonal that makes 45° with the axis of the feed line to produce perfect circular polarization. The feed line is a three-stage microstrip line that acts as an impedance transformer for perfect matching of the antenna impedance to $50\,{\Omega\:}$ at $28\:\text{G}\text{H}\text{z}$. Development of the optimum design of the printed antenna is described in detail starting with a square patch that is subjected to progressive geometrical modifications in the design phases. The objective is to reach the final design of the patch that gives a reflection coefficient magnitude of less than $-30\:\text{d}\text{B}$ and an axial ratio of less than 0.2 dB at 28 GHz.

The paper is organized as follows. Section 2 gives an explanation of the design of the proposed antenna. Section 3 presents the most important parameter sweeps as examples for the parametric study to achieve the best antenna design. Section 4 introduces an approximate equivalent circuit model for the proposed antenna. Section 5 gives an explanation of the methods used for experimental assessment of the proposed CP antenna including the antenna fabrication. Section 6 presents the surface current distribution to explain the mechanism of producing circular polarization. Section 7 demonstrates the results concerned with the far field quantities. Section 8 provides some comparisons with recently published work of similar concerns. Finally, the most important conclusions are summarized in Sect. 9.

## Printed Antenna Design

**Fig. 1:**
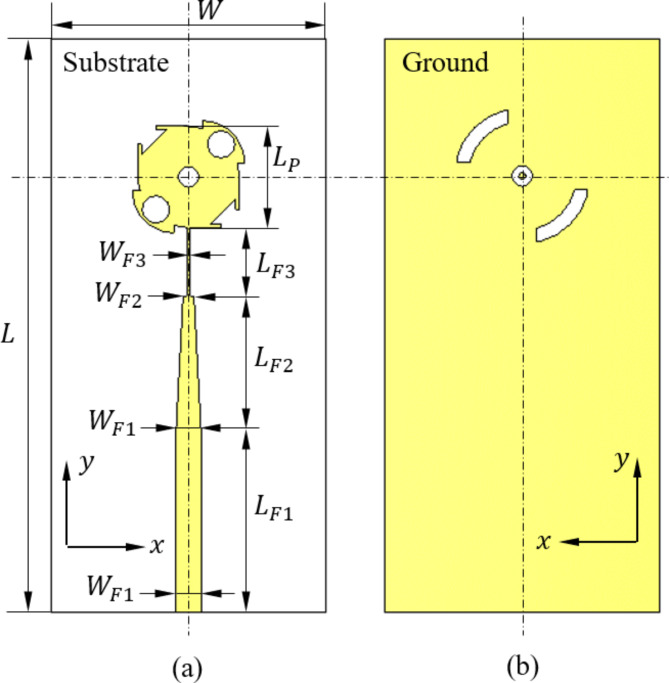
Circularly-polarized (CP) printed patch. (a) Upper surface. (b) Lower surface.

**Fig. 2:**
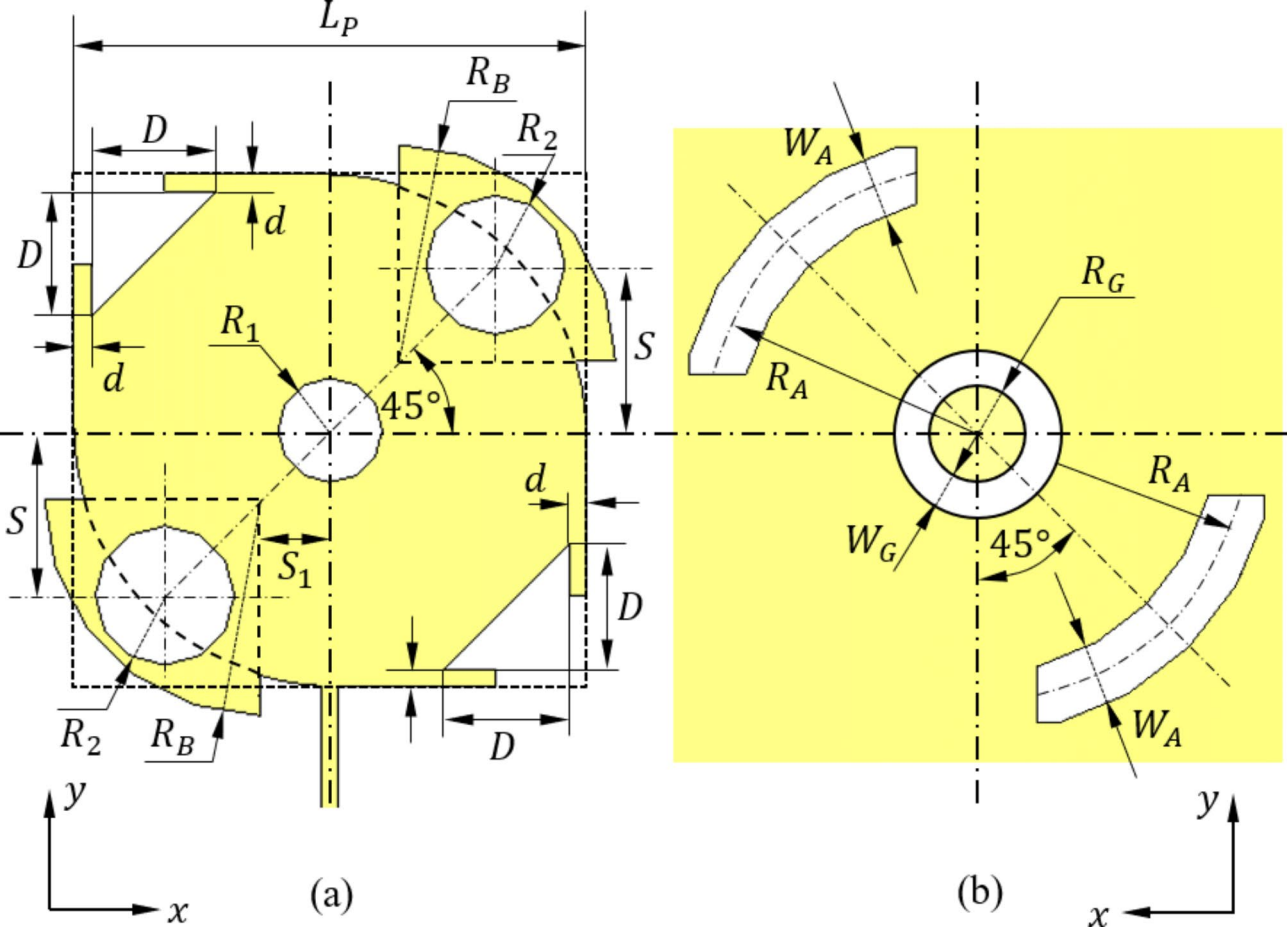
Zoomed view showing the geometry and the design parameters of (a) the patch and (b) the DGS.

The designed antenna can be regarded as a square microstrip printed patch of modified geometry. The patch structure has 45°-diagonal of symmetry as shown in Fig. 1 to produce circular polarization. Two diagonally facing corners of the square patch are blended with blending radius ${R}_{B}$. At each of these corners, a quarter-circular patch is merged to the patch. The other corners are cut as shown in Fig. 2. Three holes are perforated in the patch keeping the same 45°-diagonal symmetry. The antenna is excited by a multi-stage microstrip transmission line of tapered structure to match the antenna impedance to $50\,{\Omega\:}$. This method of impedance matching is used to avoid disturbing the geometrical symmetry of the radiating patch as in the traditional inset feed. The feeding transmission line is constructed as a three-stage microstrip line. The first stage is a uniform microstrip line of length ${L}_{F1}$ and width ${W}_{F1}$ that is constant along its length. The second stage of the feeding line is a tapered microstrip line of length ${L}_{F2}$; the width varies linearly from ${W}_{F1}$ to ${W}_{F2}$ along its length. The third stage of the feeding line is a narrow microstrip line of length ${L}_{F3}$ and width ${W}_{F3}$ that is fixed along its length. This structure of the feeding line allows impedance matching of the designed antenna at $28\:\text{G}\text{H}\text{z}$ without violating the geometrical symmetry of the radiating patch about the 45°-diagonal. Such geometrical symmetry of the resonant patch allows the excitation of two degenerate modes at $28\:\text{G}\text{H}\text{z}$ with orthogonal directions of surface current on the patch. One of these modes has even symmetry about the printed patch diagonal making 45°, and the second mode has odd symmetry about this diagonal. The modifications of the original square printed patch geometry described above are made to get the orthogonally-directed surface currents of the two degenerate modes excited at resonance ($28\:\text{G}\text{H}\text{z}$) of almost equal magnitudes. This results in two orthogonal components of the radiated electric field in the far zone with almost equal magnitudes and 90° phase shift. This mechanism of generating circular polarization is numerically investigated through simulation to demonstrate the progressive rotations of patch surface current with time to make 360° rotation over the cycle of the sinusoidal wave.

For further improvement of the circular polarization at $28\:\text{G}\text{H}\text{z}$, a DGS is used. The geometry of the DGS is also symmetric about the line making $45^\circ$ with vertical line. The DGS has a central annular slot and two diagonally facing arc slots. Such a geometry of the DGS helps to get the AR close to unity (i.e. close to $0\:\text{d}\text{B}$).

**Table 1:** Design parameters of the CP antenna of this work.

Parameter	$L$	$W$	${L}_{P}$	$D$	$d$	$S$	${S}_{1}$	${R}_{1}$	${R}_{2}$	${R}_{B}$
Value (mm)	$16.7$	$8$	$2.925$	$0.72$	$0.1$	$0.95$	$0.4$	$0.15$	$0.4$	$1.24$
Parameter	${R}_{G}$	${W}_{G}$	${R}_{A}$	${W}_{A}$	${W}_{F1}$	${L}_{F1}$	${W}_{F2}$	${L}_{F2}$	${W}_{F3}$	${L}_{F3}$
Value (mm)	$0.19$	$0.1$	$1.82$	$0.44$	$0.77$	$4.35$	$0.32$	$6.35$	$0.1$	$2.0$

## Parametric Study for Antenna Design optimization

A complete extensive parametric study based on parameter sweeps for all the geometrical design parameters presented in Figs. 1 and 2 has been performed to obtain the optimal design. Some examples of such parametric sweeps are presented and discussed. The objective of the parametric study is to enhance the antenna performance regarding the operation within a frequency band around $28\:\text{G}\text{H}\text{z}$ that is wide enough to realize prefect impedance matching and high purity of circular polarization. Thus, the objective of the parametric study is to minimize $\left|{S}_{11}\right|$ and AR within the widest possible frequency band around $28\:\text{G}\text{H}\text{z}$. The design parameters selected as examples for the parametric study are the width of the first stage of the microstrip line feeder (${W}_{F1}$), the length of tapered section of the feed line (${L}_{F2}$), the length of the arm of the patch square (${L}_{P}$), the corner holes radius (${R}_{2}$), the offset shift of the corner holes ($S$), the radius of annular opening in the ground (${R}_{G}$), and the radius of the arc-shaped opening in the ground (${R}_{A}$). In the remainder of this section, the results of the parametric sweeps of the above seven parameters are presented and discussed.

### Design parameters of the feed line

**Fig. 3:**
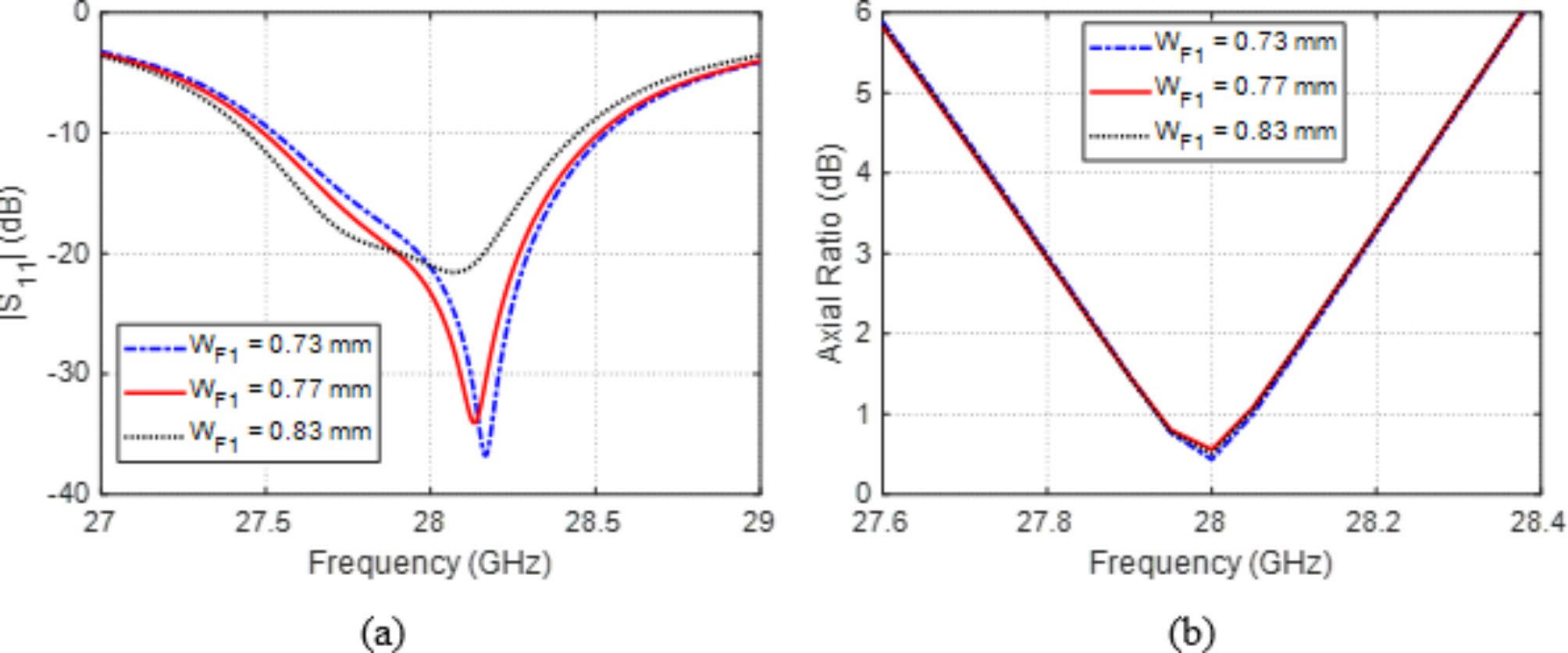
Variation effect of width, ${W}_{F1}$, of first design stage of the microstrip line on (a) $\left|{S}_{11}\right|$ and (b) AR.

The three-stage microstrip line feeder is used as an impedance transformer to set the input impedance of the antenna to $50\:{\Omega\:}$. The first stage of this feeder is a uniform microstrip line of width ${W}_{F1}$, which is constant along its length. The effect of changing ${W}_{F1}$ on the self-coupling magnitude, $\left|{S}_{11}\right|$, is demonstrated in Fig. 3a, and its effect on the AR is shown in Fig. 3b. It is shown that $\left|{S}_{11}\right|$ depends on this parameter, whereas the AR is almost independent of ${W}_{F1}$. The widest frequency band within which the patch impedance gets to $50\:{\Omega\:}$ ($\left|{S}_{11}\right|<-10\:\text{d}\text{B}$) is obtained, when ${W}_{F1}=0.77\:\text{m}\text{m}$.

**Fig. 4:**
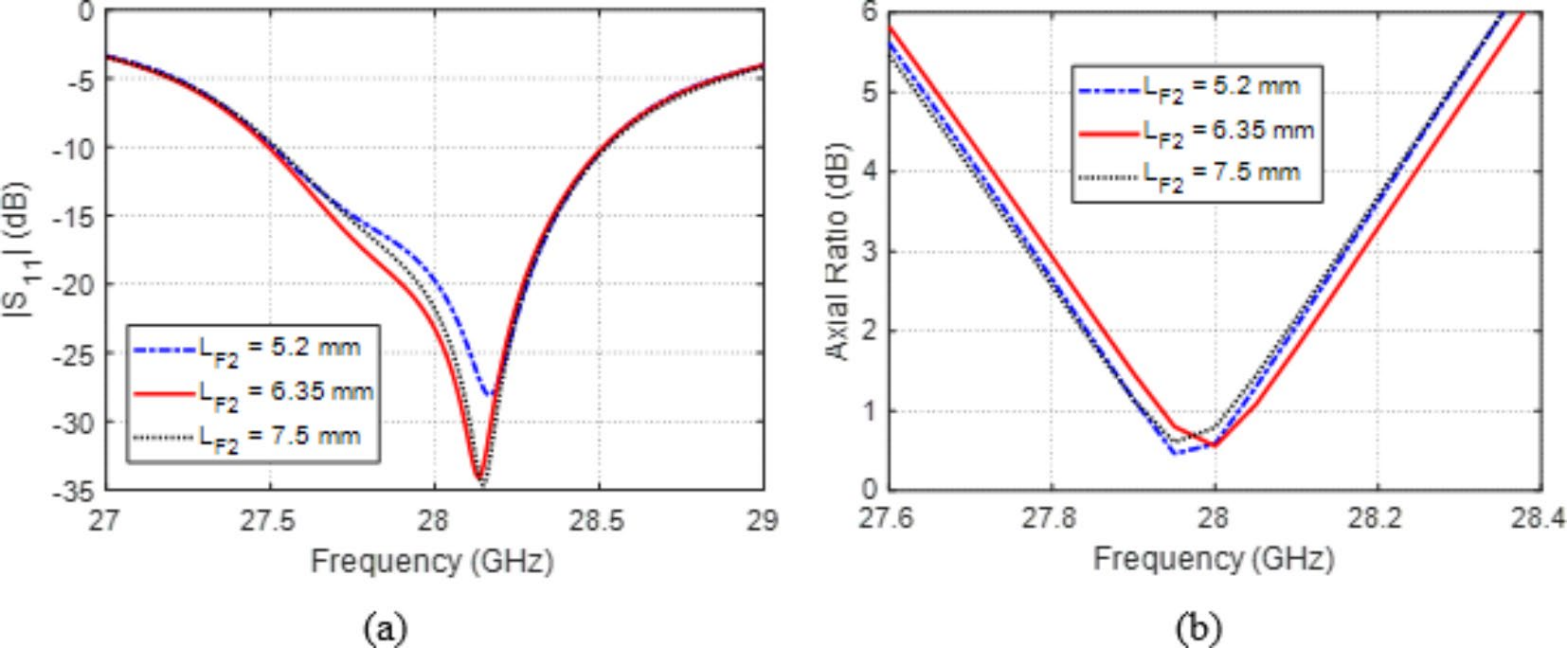
Variation effect of the length, ${L}_{F2}$, of the tapered section of the feed line on (a) $\left|{S}_{11}\right|$ and (b) AR.

The tapered section of the three-stage feeding microstrip line has a major role in antenna impedance matching. The length of this stage of the feed line controls the taper rate ($${WF}_{1}-{W}_{F2})/{L}_{F2}$$. Varying $${L}_{F2}$$ affects the frequency dependencies of $$\left|{S}_{11}\right|$$ and AR as illustrated in Fig. 4. The impedance matching is considerably affected by varying $${L}_{F2}$$ as shown in Fig. 4a. On the other hand, the AR seems to be less sensitive to varying $${L}_{F2}$$ as shown in Fig. 4b. The optimum behavior of the antenna from the perspectives of matching the antenna input impedance with the exciting feedline and the circular polarization with a center of $$28\:\text{G}\text{H}\text{z}$$ is obtained, when $${L}_{F2}=8.35\:\text{m}\text{m}$$.

### Design parameters of the Patch

Since the patch is the radiating element, the frequency at which the antenna resonates, the bandwidth at which the antenna input impedance is matched, and the 3dB AR bandwidth are drastically affected by the dimensions of the patch.

**Fig. 5:**
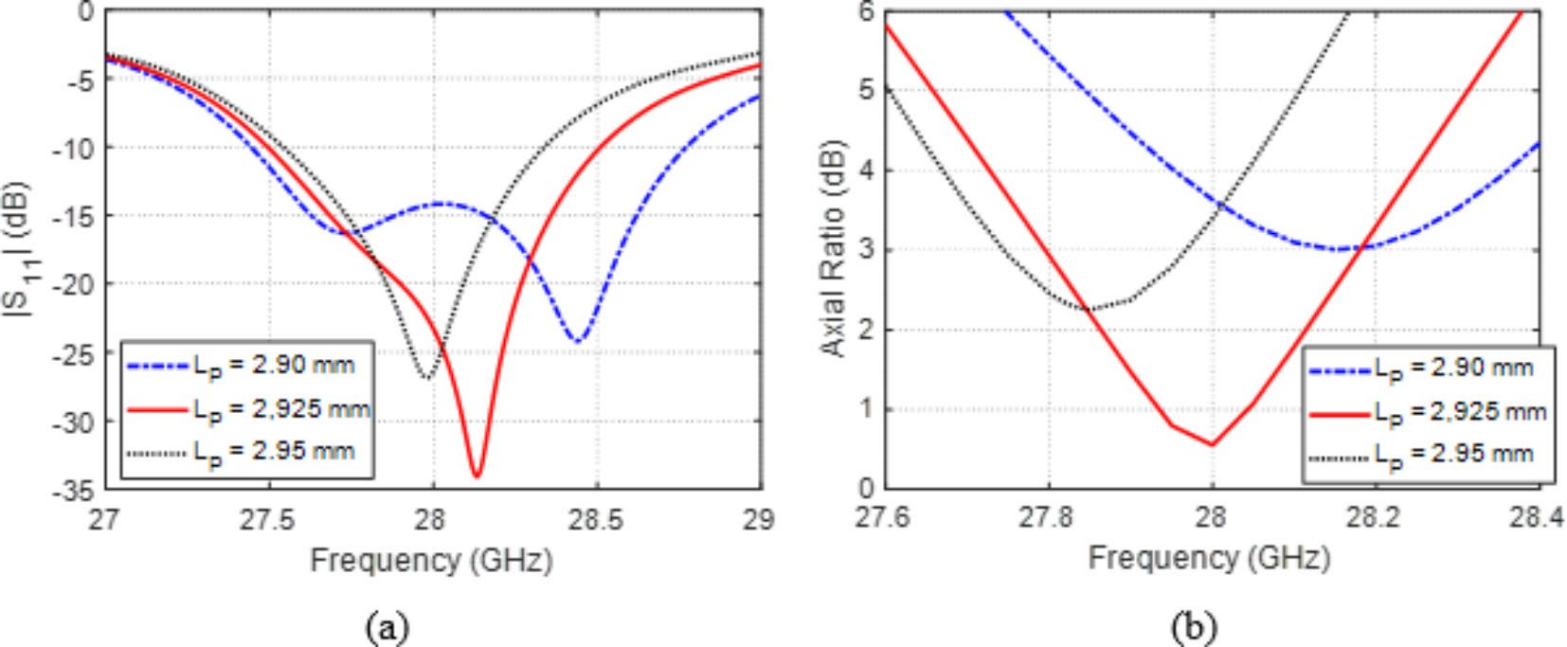
Variation effect of the length, ${L}_{P}$, of the patch on (a) $\left|{S}_{11}\right|$ and (b) AR.

The side length ${L}_{P}$ of the square patch has major effects on both $\left|{S}_{11}\right|$ and AR as illustrated in Fig. 5. The antenna resonance is very sensitive to varying ${L}_{P}$ as shown in Fig. 5a. Also, the AR is drastically affected by ${L}_{P}$ as shown in Fig. 5b. It is demonstrated that the largest bandwidth for which the input impedance is matched to the feedline has the best value when ${L}_{P}=2.9\:\text{m}\text{m}$, whereas the largest AR bandwidth ($\text{A}\text{R}<3\:\text{d}\text{B}$) is obtained when ${L}_{P}=2.925\:\text{m}\text{m}$. So, it is decided to set ${L}_{P}=2.925\:\text{m}\text{m}$.

**Fig. 6:**
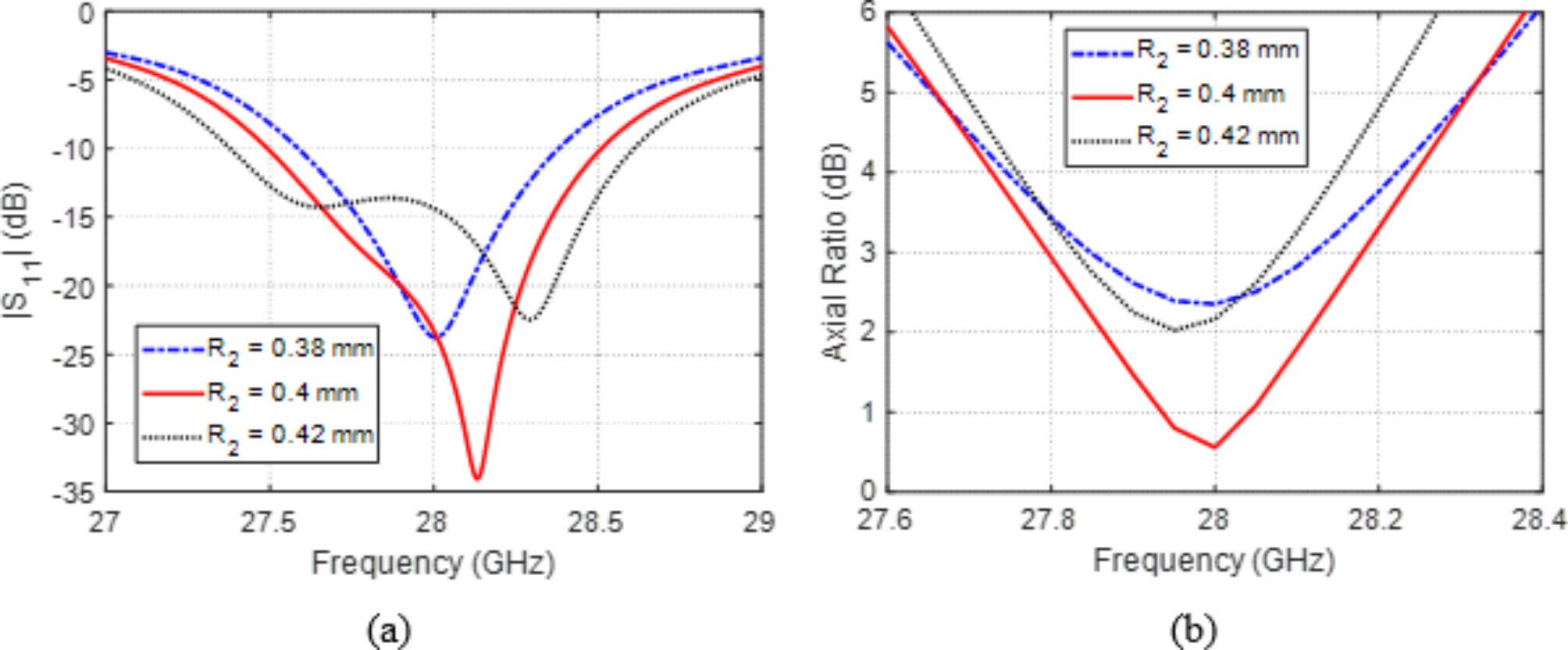
Variation effect of the radius, ${R}_{2}$, of the corner holes on (a) $\left|{S}_{11}\right|$ and (b) AR.

The radius ${R}_{2}$ of the circular holes at the corners of the patch has a major impact on both $\left|{S}_{11}\right|$ and AR as shown from the variations of the frequency responses of these performance parameters with ${R}_{2}$, which are presented in Fig. 6a and b, respectively. By setting ${R}_{2}=0.4\:\text{m}\text{m}$, the center frequency of impedance matching is set exactly at $28\:\text{G}\text{H}\text{z}$ and the best AR is also achieved at $28\:\text{G}\text{H}\text{z}$.

**Fig. 7:**
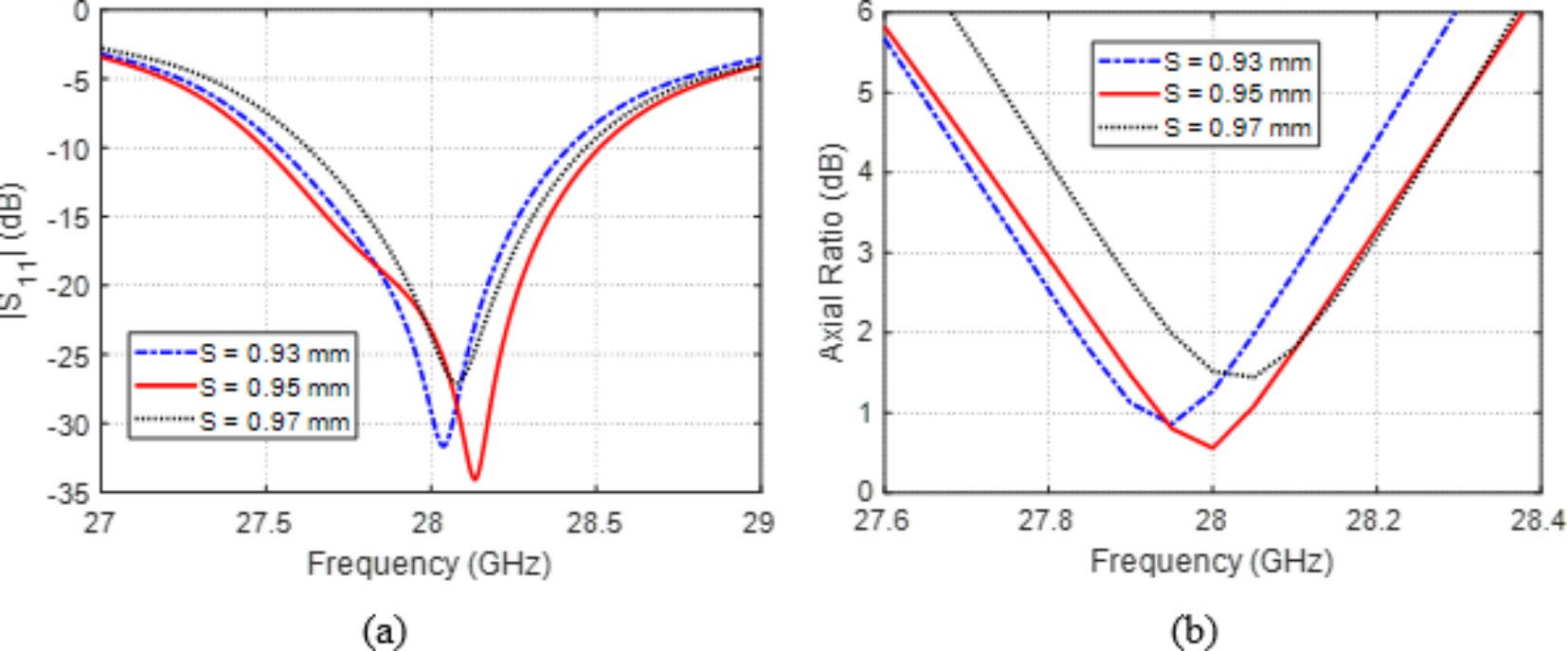
Variation effect of the offset distance, $S$, of the centers of corner holes on (a) $\left|{S}_{11}\right|$ and (b) AR.

Varying the offset distance, $S$, of the centers of corner holes from the patch center has the effects presented in Fig. 7a on $\left|{S}_{11}\right|$ within the band of $27-29\:\text{G}\text{H}\text{z}$. On the other hand, such variations affect the AR within the band of $27.6-28.4\:\text{G}\text{H}\text{z}$ as illustrated in Fig. 7b. The locations of the corner holes have major impact on both $\left|{S}_{11}\right|$ and AR as they control the current distribution on the patch. To get the best impedance matching and the most perfect circular polarization at $28\:\text{G}\text{H}\text{z}$, this design parameter should be set as $S=0.95\:\text{m}\text{m}$.

### Design parameters of the DGS

**Fig. 8:**
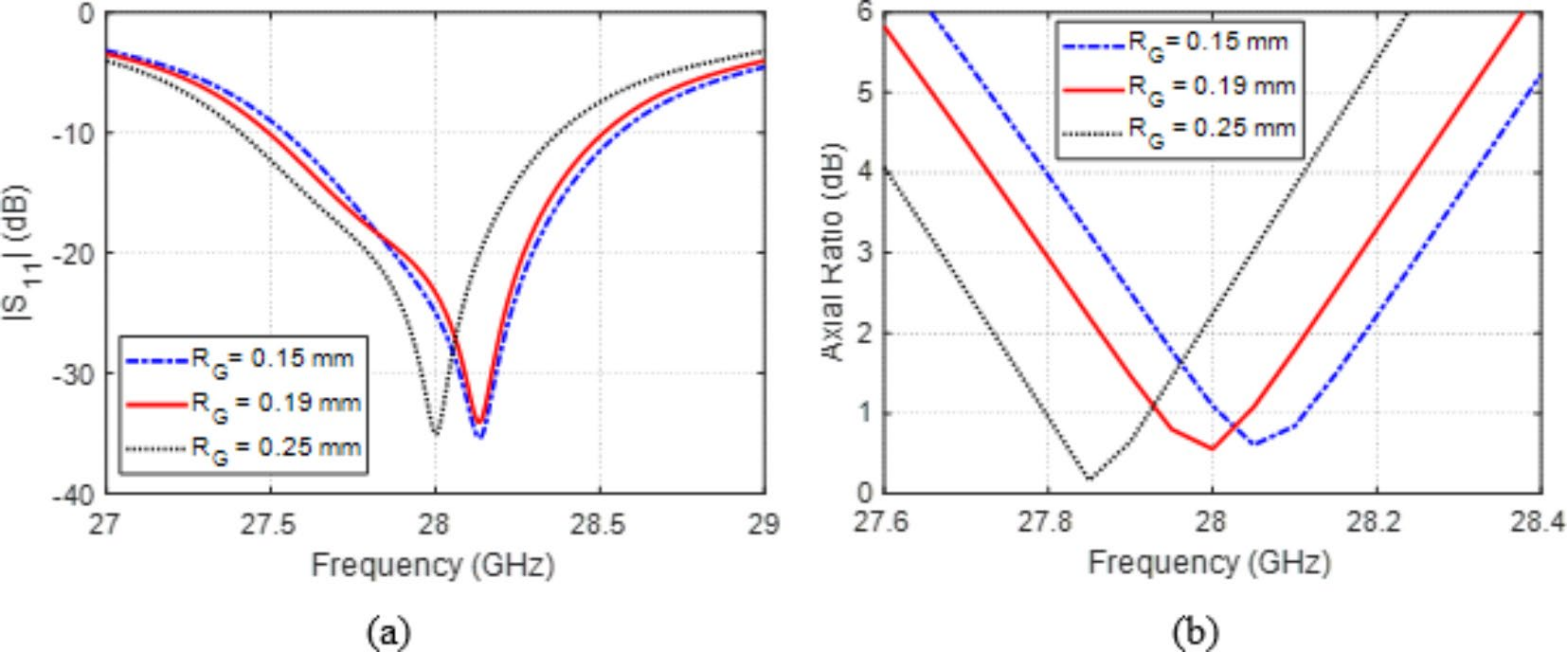
Variation effect of the radius, ${R}_{G}$, of the annular slot in the ground on (a) $\left|{S}_{11}\right|$ and (b) AR.

Varying the radius, ${R}_{G}$, of the annular slot in the ground plane that lies just below the center of the radiating patch affects the frequency responses of $\left|{S}_{11}\right|$ and AR as demonstrated in Fig. 8. The best value for ${R}_{G}$ is $0.19\:\text{m}\text{m}$, which results in the largest frequency bandwidth, where the antenna input impedance is matched to the feed line impedance and the most perfect circular polarization.

**Fig. 9:**
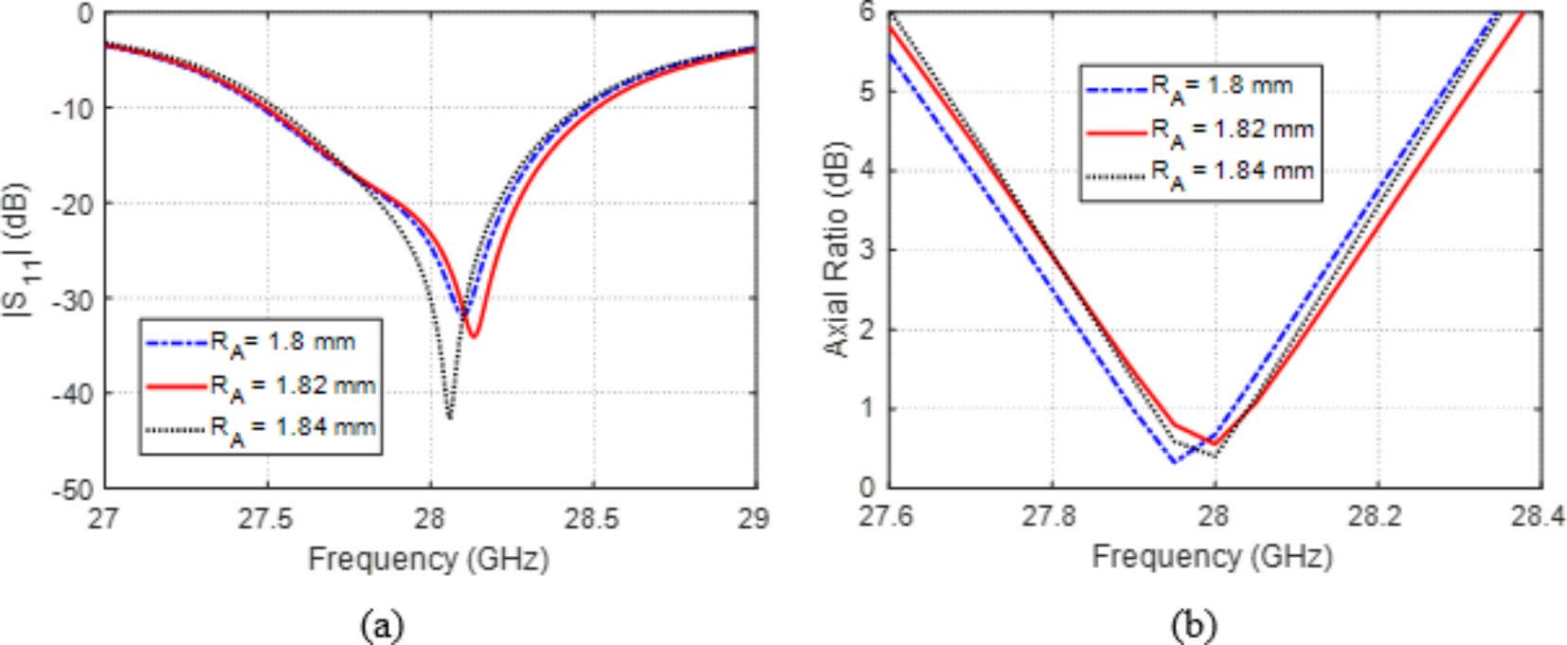
Variation effect of the radius, ${R}_{A}$, of the arc slots in the ground on (a) $\left|{S}_{11}\right|$ and (b) AR.

The two arc slots in the DGS have major contribution to producing the perfect circular polarization of the antenna, as they help to establish the geometric symmetry about the $45^\circ$-diagonal as shown in Figs. 1 and 2. Varying the radius, ${R}_{A}$, of the arc rings in the ground plane leads to varying the $\left|{S}_{11}\right|$ and AR versus frequency as illustrated in Fig. 9a and b, respectively. It is shown that setting ${R}_{A}\:=1.82\:\text{m}\text{m}$ results in the widest frequency band with $\left|{S}_{11}\right|<-10\:\text{d}\text{B}$ and $\text{A}\text{R}<3\:\text{d}\text{B}$. Also, this value of ${R}_{A}$ leads to centralization of both frequency bands exactly at $28\:\text{G}\text{H}\text{z}$.

It should be noted that a complete parametric study is performed to obtain the best estimate of the geometrical design parameters shown in Figures l and 2. These values are given in Table 1.

## Equivalent Circuit Model of the proposed antenna

**Fig. 10:**
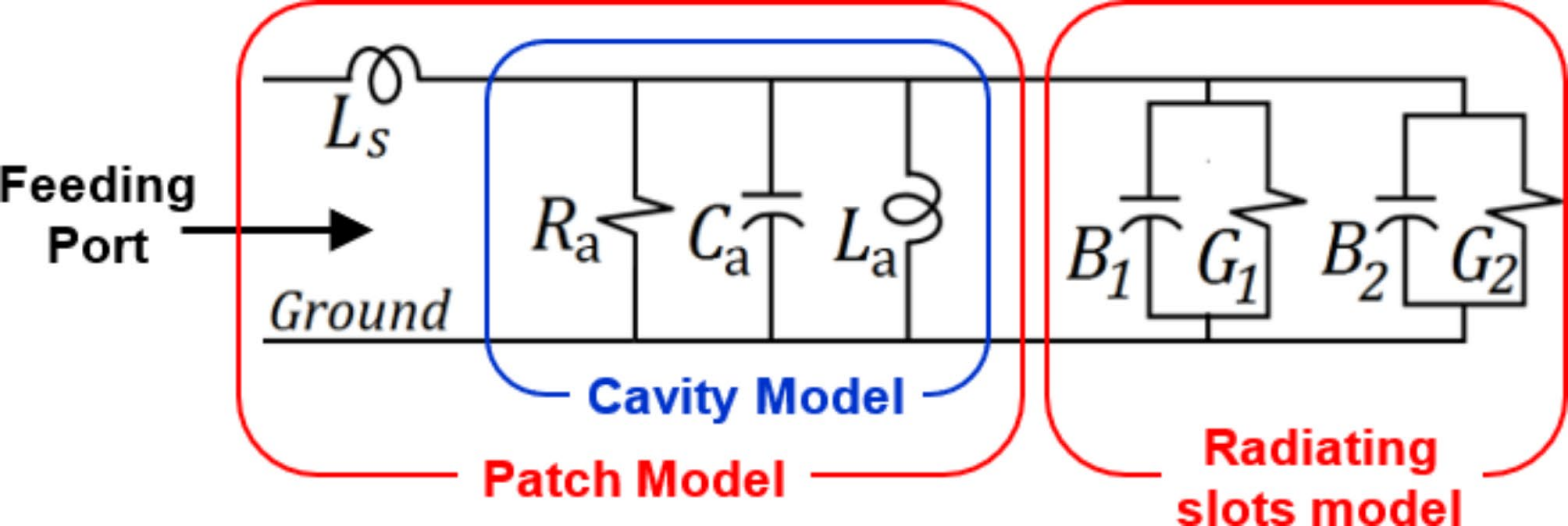
Equivalent circuit model of the primary patch presented in Fig. 1.

An equivalent circuit model of the primary patch of the proposed antenna has been deduced using the transmission line model in a way similar to that followed in^8,9^. The patch circuit model is shown in Fig. 10. The cavity subtended between the main patch and the ground plane can be modeled using the ${R}_{a}$, ${L}_{a}$, ${C}_{a}$ elements as shown in Fig. 10. The patch resonance is calculated as follows:

1 $${f}_{0}=\frac{1}{2\pi\:\sqrt{{L}_{a}{C}_{a}}}$$

At this frequency, the reactance of ${L}_{a}$ cancels that of ${C}_{a}$. The series inductance ${L}_{s}$ is added to the circuit model to account for the non-zero thickness of the patch. Thus, at resonance, the input impedance of the patch can be expressed as follows:

2 $${Z}_{\text{i}\text{n}}={R}_{a}+j2\pi\:{f}_{0}{L}_{s}$$

In the equivalent circuit model, the conductances ${G}_{1}$ and ${G}_{2}$ account for the power loss due to radiation from the first and second radiating slots, respectively. The susceptances ${B}_{1}$ and ${B}_{2}$ account for the reactive power storage in the cavity surrounded by the radiating slots.

**Fig. 11:**
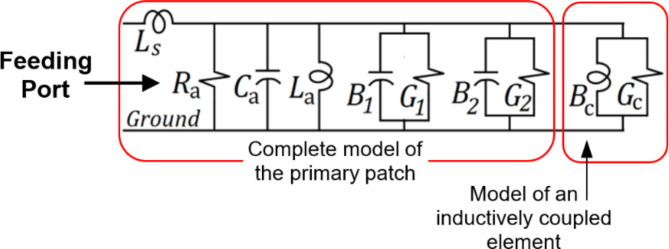
Equivalent circuit of the main patch, when loaded by inductively-coupled elements.

Two types of such loads can be used to control the locations of the resonant frequencies and to improve the impedance matching at these frequencies: inductively-loaded and capacitively-loaded elements. In the proposed design, only inductive loads are added. The equivalent circuit model of the modified patch antenna is presented in Fig. 11. The merged quarter-circuit patch can be seen as being inductively-coupled (short-circuited) to the main patch. The inductively-loaded elements can be represented by shunt coil and resistance (conductance ${G}_{c}$) as shown in the circuit model. This load can be used for tuning the resonant frequencies, and, in the meantime, to control the antenna impedance at these resonances.

## Fabrication and practical measurements

For experimental evaluation of the designed antenna performance, a prototype is fabricated and subjected to measuring the reflection coefficient over the band of 27–29 GHz, gain, AR, and radiation efficiency over the band of $27.5-28.5\:\text{G}\text{H}\text{z}$. The simulation results are verified by practical measurements.

### Antenna fabrication

**Fig. 12:**
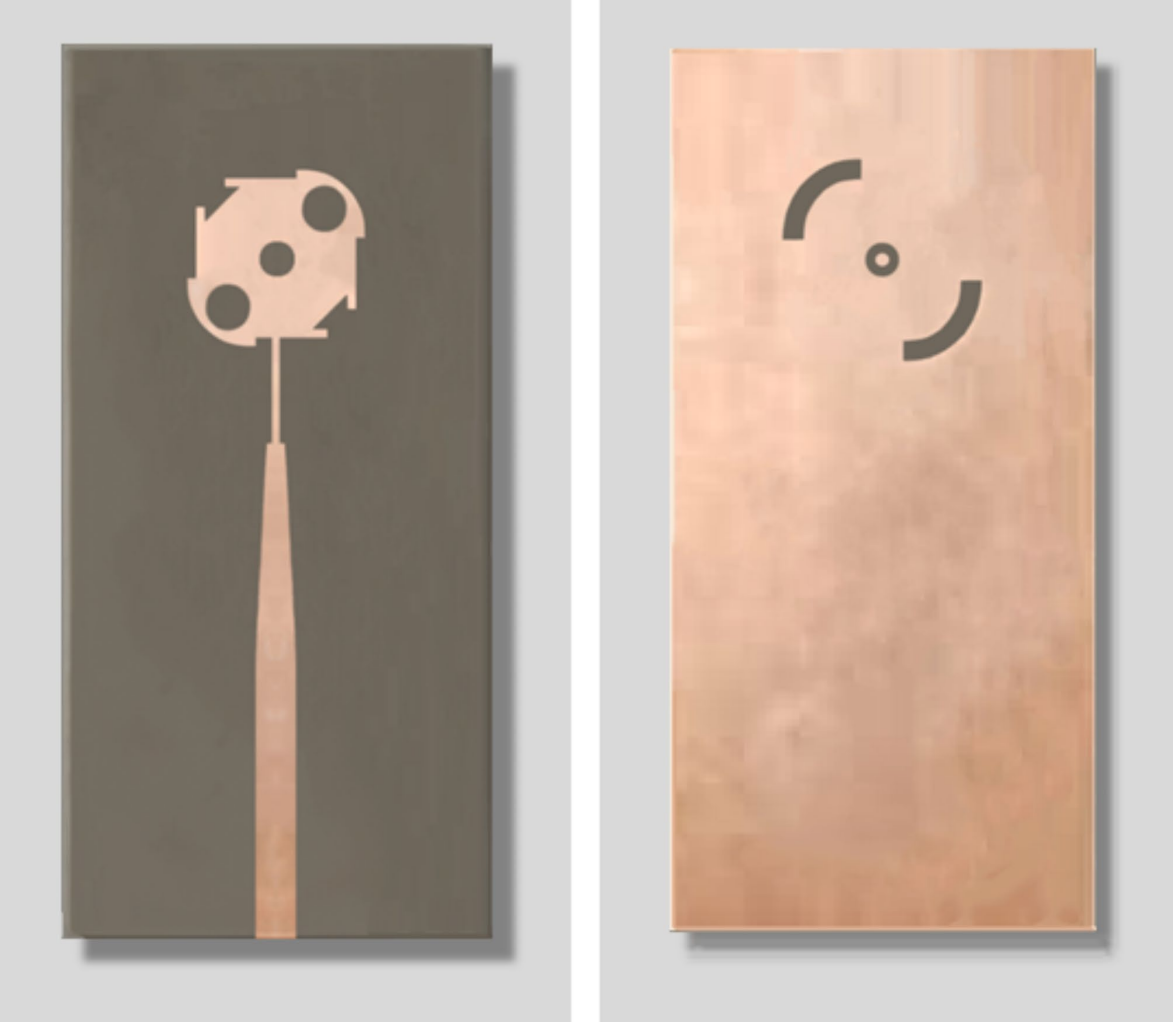
CP antenna prototype (a) Top, and (b) Bottom, view.

The proposed antenna is fabricated on a double-sided substrate of RO-4003 C material of height, $h=0.25\:\text{m}\text{m}.$ The fabrication is performed by applying lithography on both sides of the substrate. The fabricated antenna is shown in Fig. 12.

##  Measurement of the Antenna Reflection coefficient

**Fig. 13:**
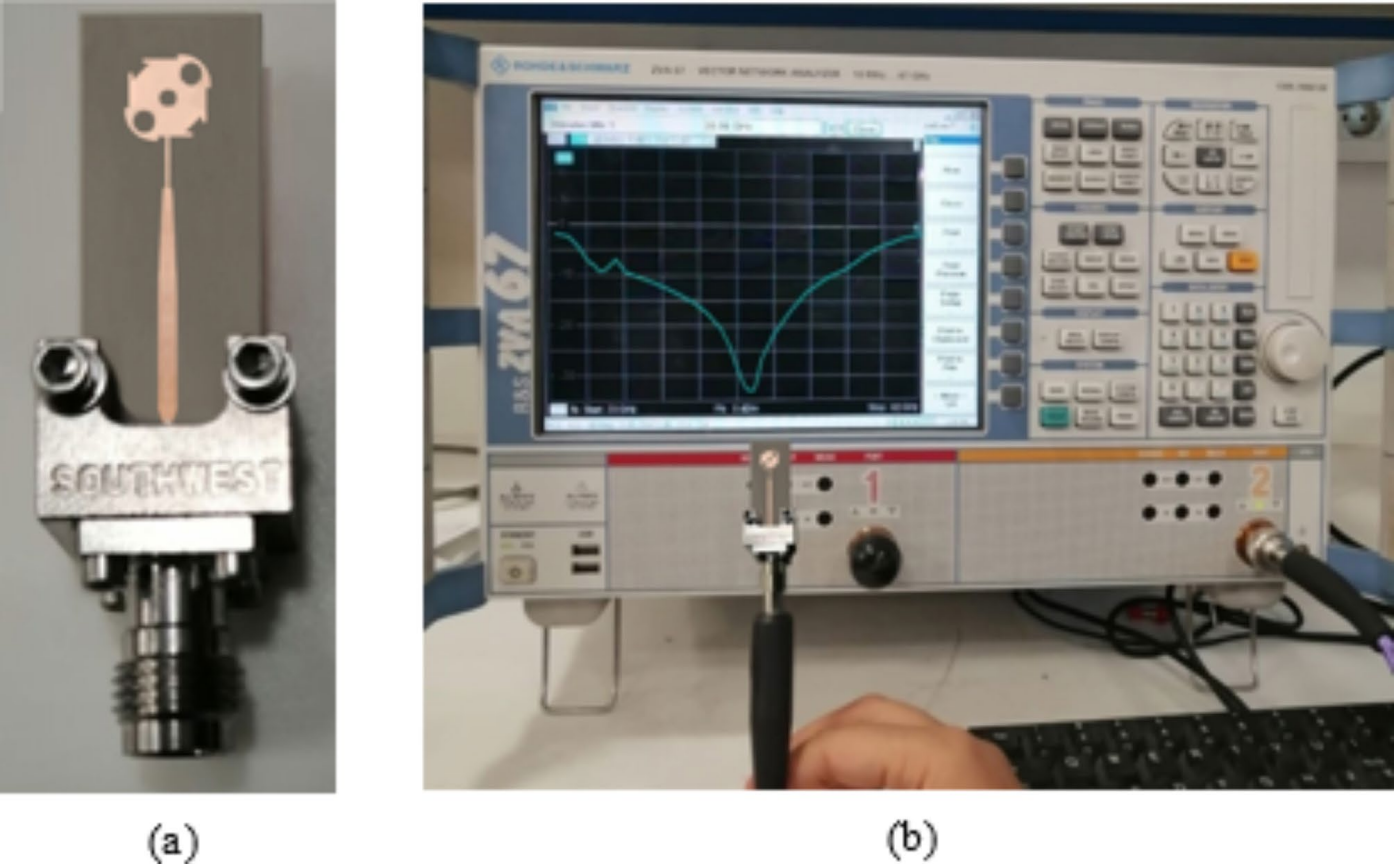
The end-launch connector mounted to the fabricated antenna and connected to the VNA of model ZVA-67 for measuring $\left|{S}_{11}\right|$. (a) Front view of the antenna. (b) Measurement of $\left|{S}_{11}\right|$.

**Fig. 14:**
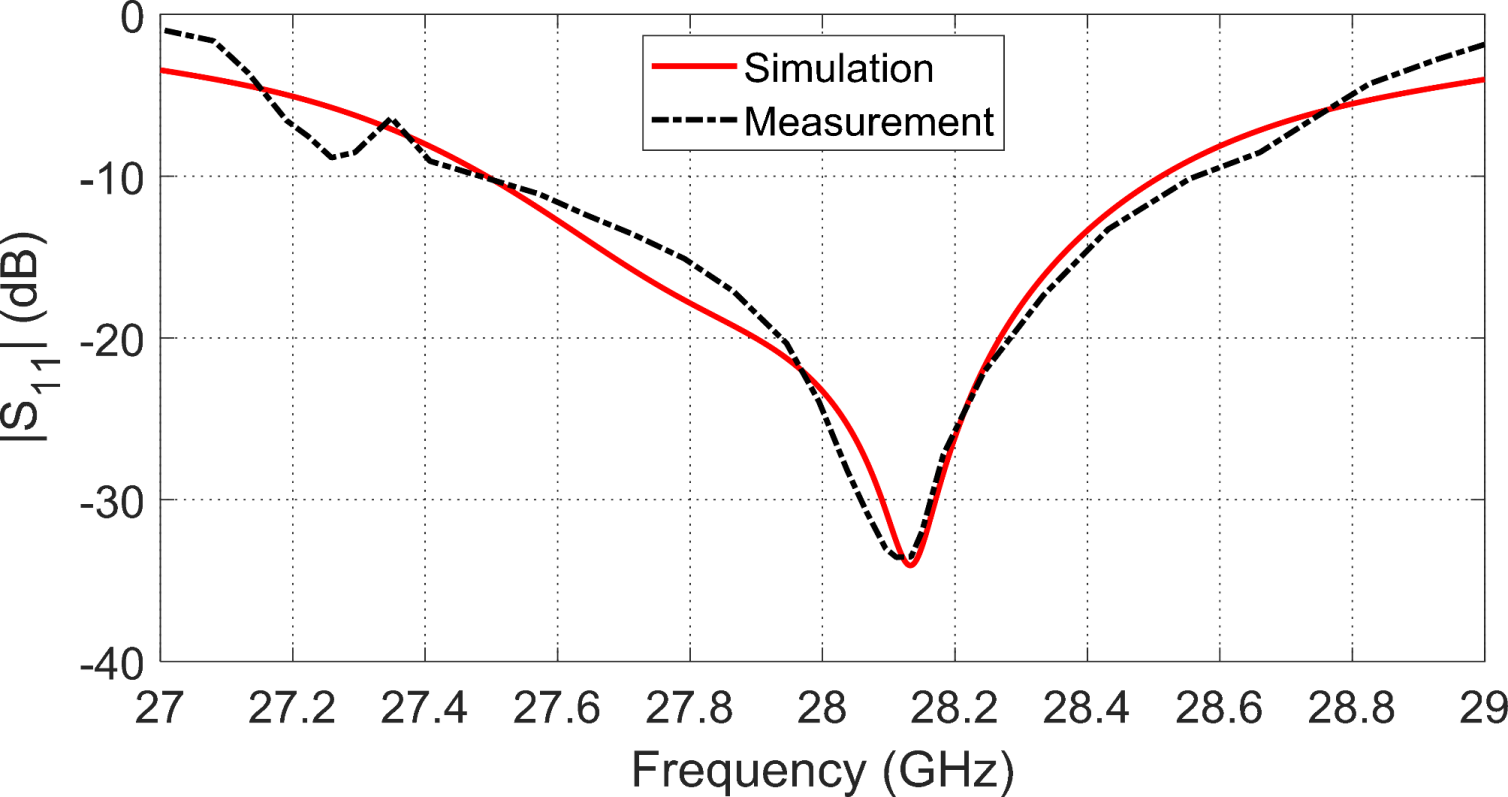
Magnitude of $\left|{S}_{11}\right|$ of the proposed antenna.

The vector network analyzer (VNA) of model Rhode and Schwartz ZVA67 is employed to measure $\left|{S}_{11}\right|$ at the antenna feeding port, as shown in Fig. 12b. A coaxial launcher of type 2.4 mm is mounted to the antenna as shown in Fig. 13a, and Fig. 13b. The VNA calibration and the reflection coefficient measurement are performed in the band of $27-29\:\text{G}\text{H}\text{z}$. The measured frequency response of $\left|{S}_{11}\right|$ is presented in Fig. 14 in comparison to the results of simulation showing good agreement. The impedance matching frequency band is shown to be $27.5-28.5\:\text{G}\text{H}\text{z}$, i.e. exactly centered at $28\:\text{G}\text{H}\text{z}$.

### Measurement of the Far Field quantities

The far field of the introduced CP antenna is evaluated through the estimation of antenna gain, AR, and radiation efficiency in the form of frequency responses. These responses are compared to those obtained through simulation. The measurements over the operational frequency band are performed by applying the method described in^10,11^. In this method, port 1 of the VNA is used to connect the designed antenna, while port 2 is used for the connection of the reference antenna (RA). The measured mutual S-parameter S_21_ is used as an indicator of the dependence of gain, AR, and radiation efficiency on frequency in the range of interest. A 3D rotator is used to allow the antenna test over the whole 360^°^. The reference antenna is a high-gain antenna mounted on a rotator to enable its rotation about the line-of-sight (LoS) between the two antennas. Thus, the boresight direction of the RA is always pointing to the antenna to be tested during measurement.

#### Antenna Gain Measurement

For measuring the gain, a reference-gain antenna of known gain over the frequency band of measurement is used on VNA port 2, whereas the designed antenna is set on port 1. The method of measuring the gain of the designed antenna is described in^10,11^.

#### Radiation Efficiency Measurement

Measured values of ${S}_{21}$ are used to obtain the radiation efficiency of the antenna under test (AUT). It is necessary to know the output power during measurement. The whole radiated power is obtained by integrating the radiated power density measured on the surface of a sphere at the far zone of the antenna, and its center is at the location of the antenna. The method applied for measuring the radiation efficiency is described in^10,11^.

#### Axial ratio measurement

For measuring the AR, the polarization of the reference antenna is altered between and horizontal ($x$-direction) polarization and vertical ($y$-direction) polarization, while the AUT is being rotated. This is achieved by rolling the reference antenna 90° about its axis. In this way, the horizontally- and vertically-polarized components (${E}_{x}$ and ${E}_{y}$) of the field radiated by the AUT can be separately received by the reference antenna and measured by the VNA. The RHCP and LHCP radiated field components can be calculated from ${E}_{x}$ and ${E}_{y}$ as follows:

3-a $${E}_{L}=\frac{1}{2}\left({E}_{x}+j{E}_{y}\right)$$

3-b $${E}_{R}=\frac{1}{2}\left({E}_{x}-j{E}_{y}\right)$$

Axial Ratio (AR) is defined by the following expression:

4 $${AR}=\left|\frac{\left|{E}_{L}\right|+\left|{E}_{R}\right|}{\left|{E}_{L}\right|-\left|{E}_{R}\right|}\right|=\left|\left(\left|\frac{{E}_{L}}{{E}_{R}}\right|+1\right){\left(\left|\frac{{E}_{L}}{{E}_{R}}\right|-1\right)}^{-1}\right|$$

where ${E}_{L}$ and ${E}_{R}$ are the LHCP and RHCP electric field components. From (3), the ratio ${E}_{L}/{E}_{R}$ can be expressed as follows:

5 $$\frac{{E}_{L}}{{E}_{R}}=\left(\frac{{E}_{x}}{{E}_{y}}+j\right){\left(\frac{{E}_{x}}{{E}_{y}}-j\right)}^{-1}$$

Let us define the following quantity.

6 $${S}_{xy}\triangleq\:\frac{{E}_{x}}{{E}_{y}}=\frac{{\left.{S}_{s1}\right|}_{x}}{{\left.{S}_{s1}\right|}_{y}}$$

It has been considered that when the RA is aligned to the $x$-axis, only ${E}_{x}$ is received (i.e. ${\left.{S}_{s1}\right|}_{x}$ is measured by the VNA); and when it is aligned to $y$-axis, only ${E}_{y}$ is received (i.e. ${\left.{S}_{s1}\right|}_{y}$ is measured by the VNA).

7 $$\frac{{E}_{L}}{{E}_{R}}=\frac{{S}_{xy}+j}{{S}_{xy}-j}$$

From previous equations, the expression for ${AR}$ is reduced to the following form:

8 $${AR}=\left|\left(\left|\frac{{S}_{xy}+j}{{S}_{xy}-j}\right|+1\right){\left(\left|\frac{{S}_{xy}+j}{{S}_{xy}-j}\right|-1\right)}^{-1}\right|$$

Thus, the directional pattern of the axial ratio, ${AR}(\theta\:,\varphi\:)$, can be represented as follows.

9 $${AR}(\theta\:,\varphi\:)=\left|\frac{\left|\frac{{\left.{S}_{21}\left(\theta\:,\varphi\:\right)\right|}_{x}+j{\left.{S}_{21}\left(\theta\:,\varphi\:\right)\right|}_{y}}{{\left.{S}_{21}\left(\theta\:,\varphi\:\right)\right|}_{x}-j{\left.{S}_{21}\left(\theta\:,\varphi\:\right)\right|}_{y}}\right|+1}{\left|\frac{{\left.{S}_{21}(\theta\:,\varphi\:)\right|}_{x}+j{\left.{S}_{21}(\theta\:,\varphi\:)\right|}_{y}}{{\left.{S}_{21}(\theta\:,\varphi\:)\right|}_{x}-j{\left.{S}_{21}(\theta\:,\varphi\:)\right|}_{y}}\right|-1}\right|$$

## Surface current distribution

**Fig. 15:**
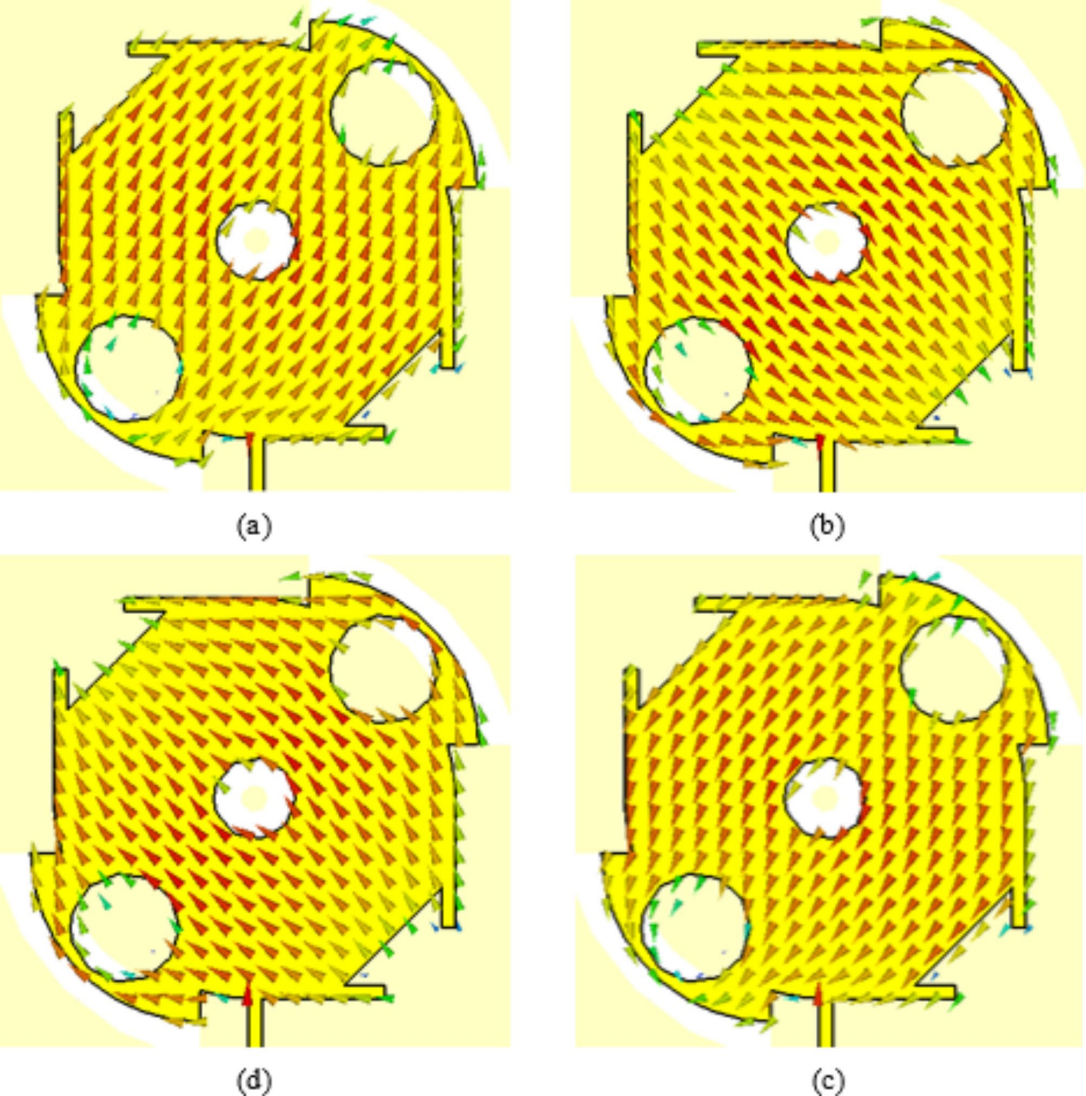
Directions of the surface current on the radiating patch at sequential (progressive) phases separated by $90^\circ$ on one cycle of the sinusoidal wave at 28 GHz. (a) Phase $0^\circ$. (b) Phase $90^\circ$. (c) Phase $180^\circ$. (d) Phase $270^\circ$.

**Fig. 16:**
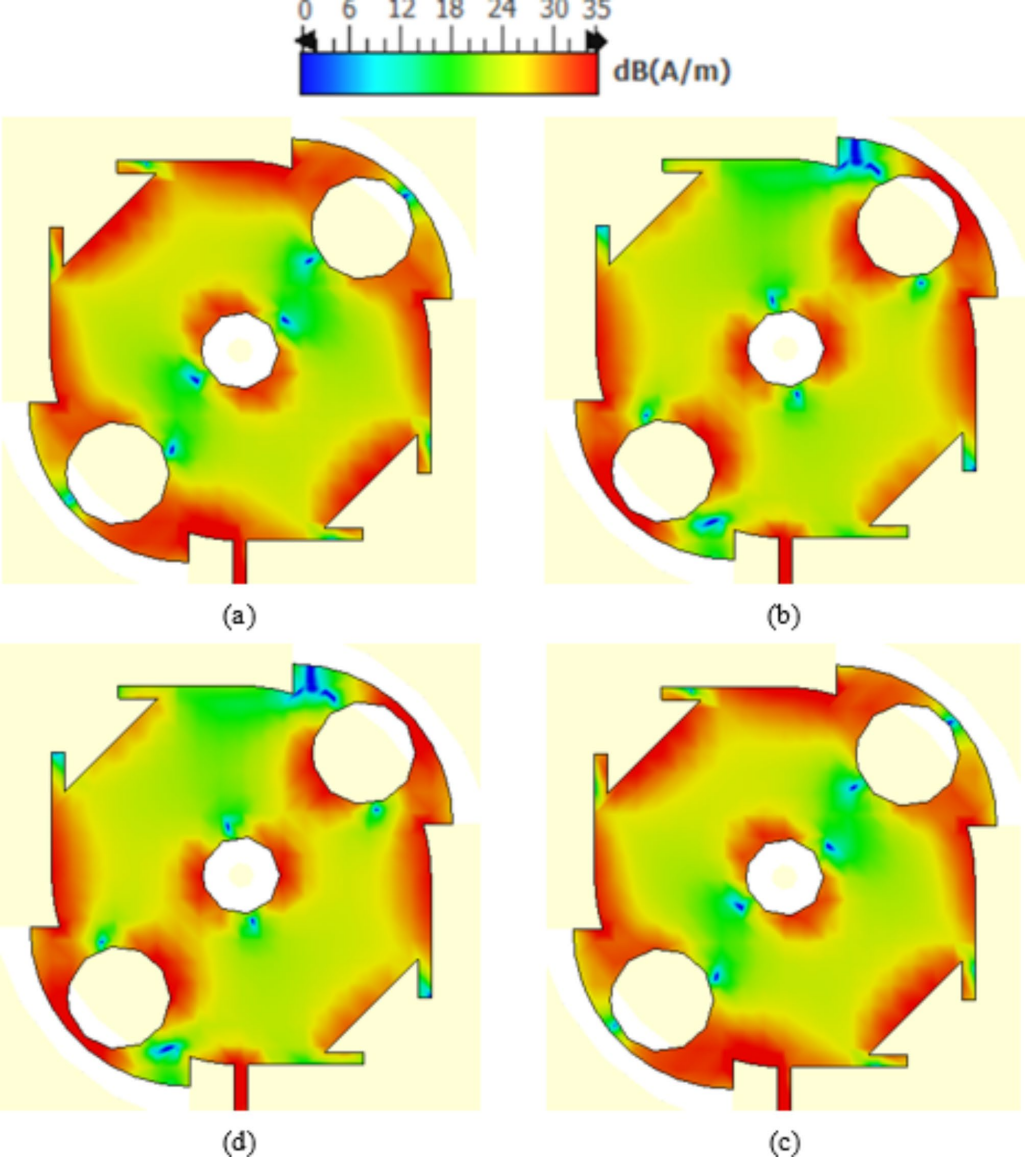
Distribution of the surface current magnitude on the radiating patch at sequential (progressive) phases separated by $90^\circ$ on one cycle of the sinusoidal wave at 28 GHz. (a) Phase angle $0^\circ$. (b) Phase angle $90^\circ$. (c) Phase angle $180^\circ$. (d) Phase angle $270^\circ$.

Both the directions and magnitudes of the current on the patch surface are distributed as shown in Figs. 15 and 16, respectively. The current directions are presented sequentially every quarter of the periodic time of the sinusoidal wave (i.e. at phases $0^\circ$, $90^\circ$, $180^\circ$ and 270°). It is shown that the arrows representing the surface current rotate 90° every one-quarter of the wave cycle. Also, it is clear that the current magnitudes corresponding to the four phases are equal, which means that the radiated field at 28 GHz has pure circular polarization. The sense of rotation of the current arrows with progressive phase is clock-wise, which indicates that the radiated wave is LHCP.

## Radiation characteristics of the designed antenna

The simulation results related to the radiation characteristics of the proposed CP antenna, including the radiation patterns, gain, AR, and radiation efficiency, are presented and discussed in this section.

### Radiation patterns

**Fig. 17:**
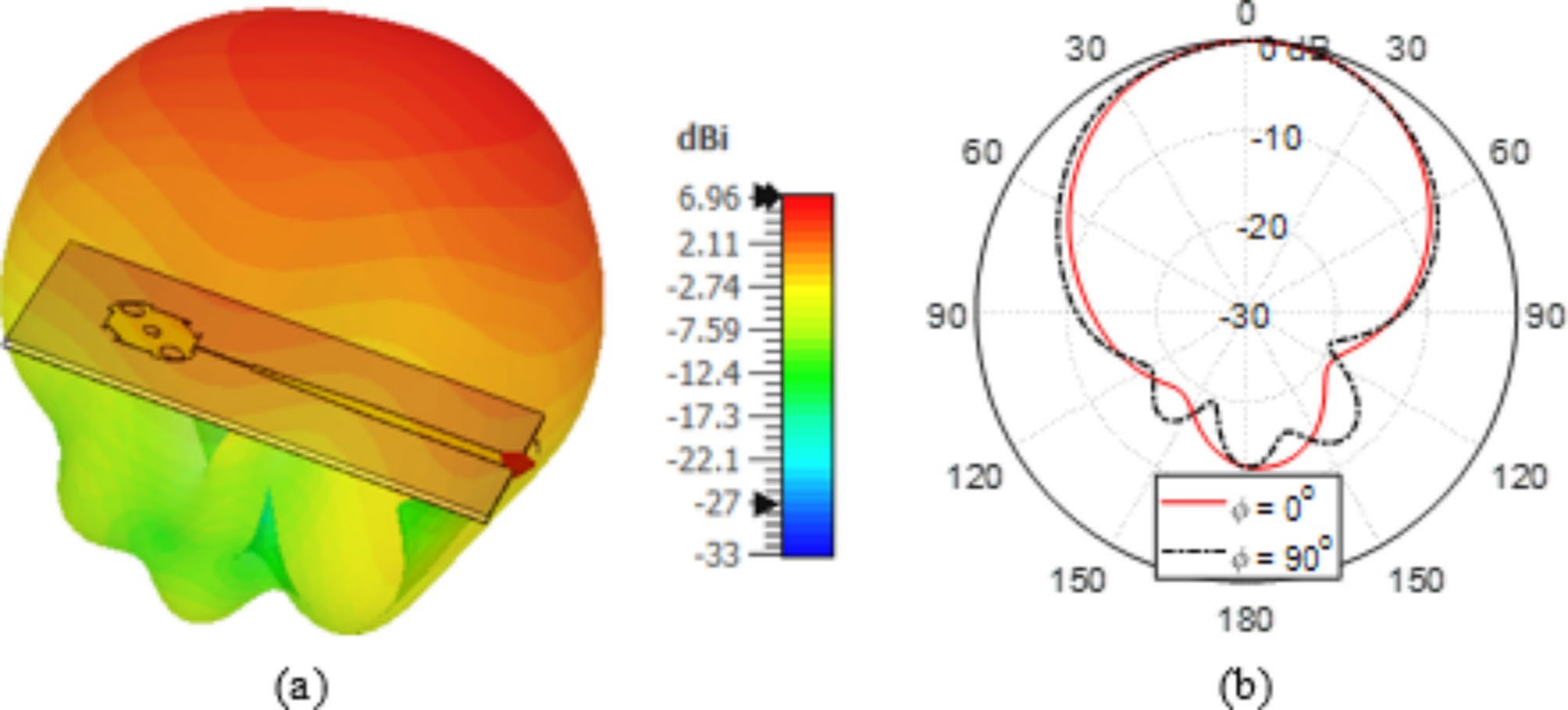
Radiation patterns of the total field produced by the proposed patch antenna in the far zone at $28\:\text{G}\text{H}\text{z}$ in the elevation plane (a) 3D radiation pattern. (b) 2D radiation patterns in the elevation plane $\varphi\:=0^\circ$ and $\varphi\:=90^\circ$.

**Fig. 18:**
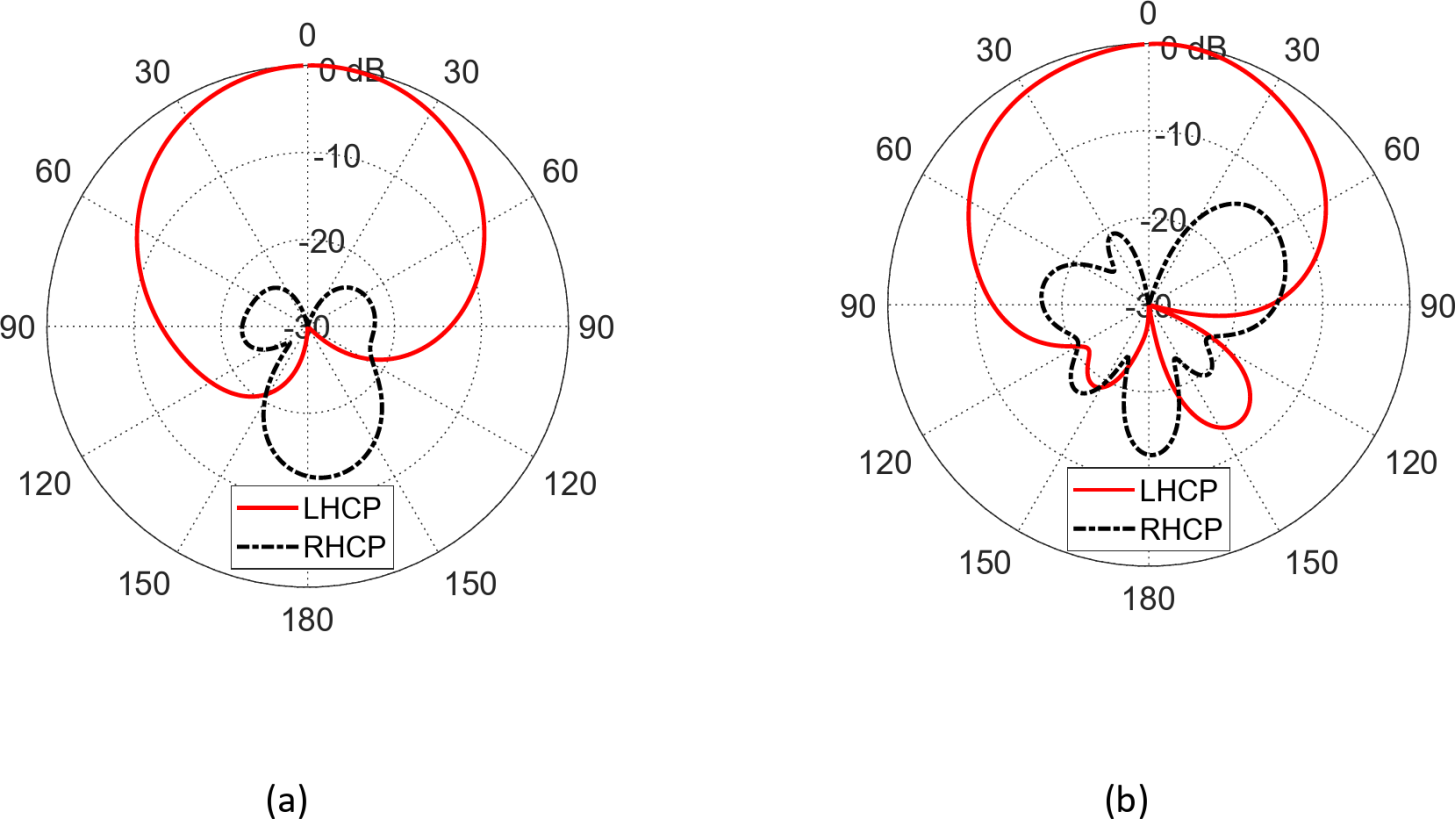
Radiation patterns of the LHCP (co-polarized) field and the RHCP (cross-polarized) field components at $28\:\text{G}\text{H}\text{z}$ in the elevation plane (a) $\varphi\:=0^\circ$ and (b) $\varphi\:=90^\circ$.

Figure 17 illustrates the radiation patterns of the whole field produced by the proposed patch antenna in the far zone at 28 GHz in the planes $\varphi\:=0^\circ$ and $\varphi\:=90^\circ$. The radiation patterns of the LHCP and RHCP fields are presented in Fig. 18. The proposed antenna produces an RHCP field as the co-polarized component with a very low level of the LHCP field as the cross-polarized component.

**Fig. 19:**
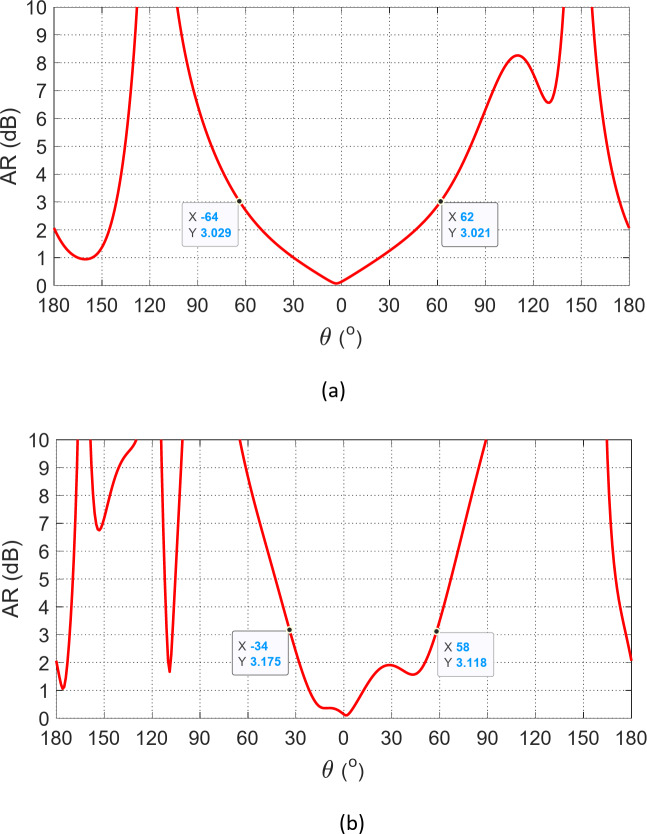
Patterns of the AR of the presented antenna in the elevation planes (a) $\varphi\:=0^\circ$ and (b) $\varphi\:=90^\circ$.

The patterns of the AR are given in Fig. 19. It is clear that the 3-dB AR beamwidth is about $124^\circ$ in the plane $\varphi\:=0^\circ$ and $92^\circ$ in the elevation plane $\varphi\:=90^\circ$. Such shapes of the patterns in the elevation planes and the beamwidth allow the proposed patch antenna to be a good candidate for arrays designed for various applications of beamforming and beam steering, and also, for MIMO systems designed with various diversity schemes including spatial and polarization diversities.

### Axial ratio variation with frequency

**Fig. 20:**
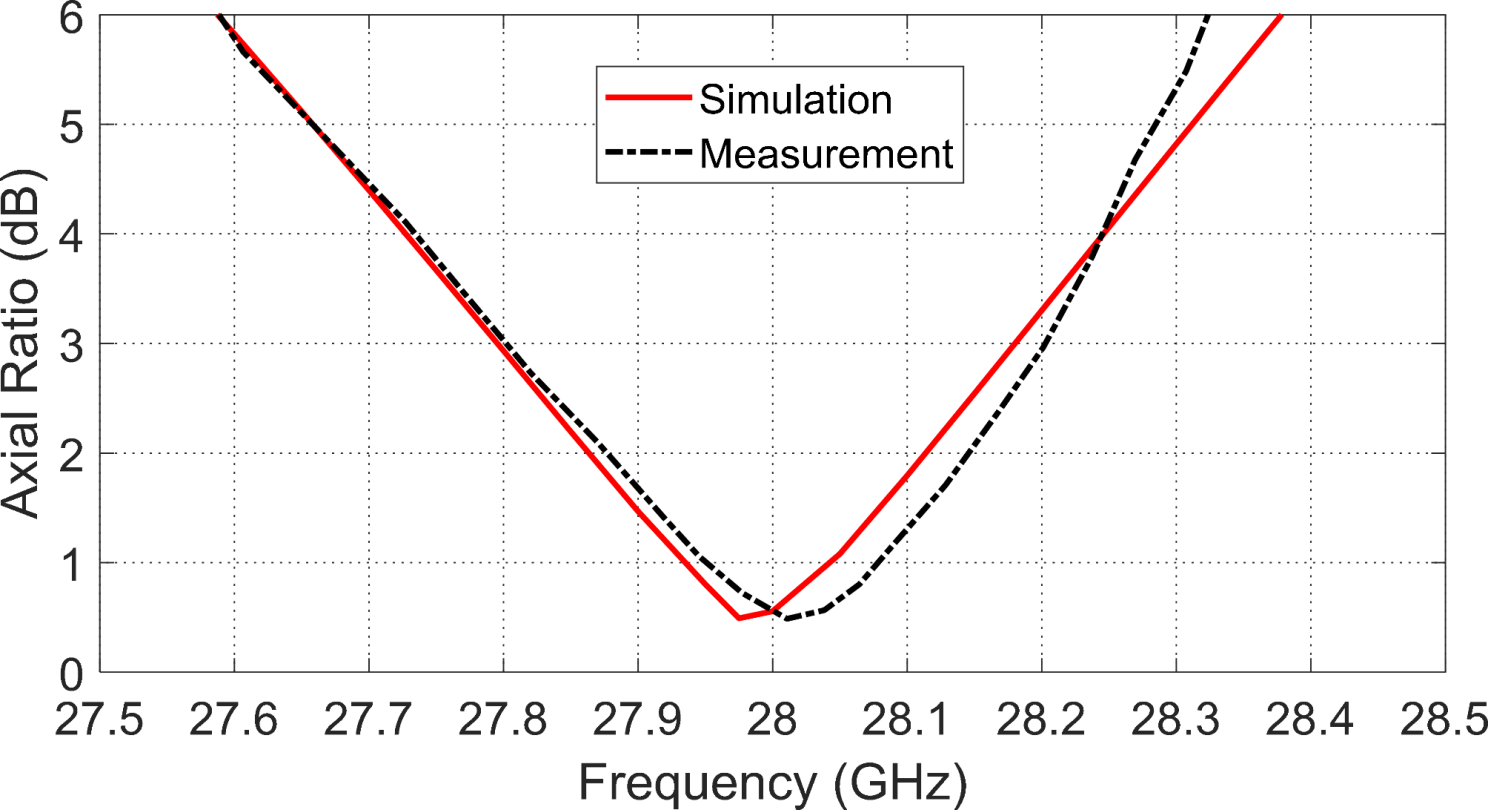
Frequency dependence of the AR of the designed CP antenna on the frequency.

The designed antenna is characterized by its perfect circular polarization within the frequency band of operation ($27.8-\:28.2\:\text{G}\text{H}\text{z}$). Figure 18 illustrates that the AR has a minimum value equal to $0.4\:\text{d}\text{B}$ at about $28\:\text{G}\text{H}\text{z}.$ Such excellent value of the AR is achieved owing to the perfect design of the antenna geometry as described above and presented in Figs. 1 and 2. The AR is measured as described previously. Figure 20 reveals that the measured frequency response of the AR agrees well with that obtained by simulation. It is clear that the AR is below $3\:\text{d}\text{B}$ over the frequency band of 27.8 to 28.2 GHz, (0.4 GHz 3-dB AR BW).

### Maximum gain variation with frequency

**Fig. 21:**
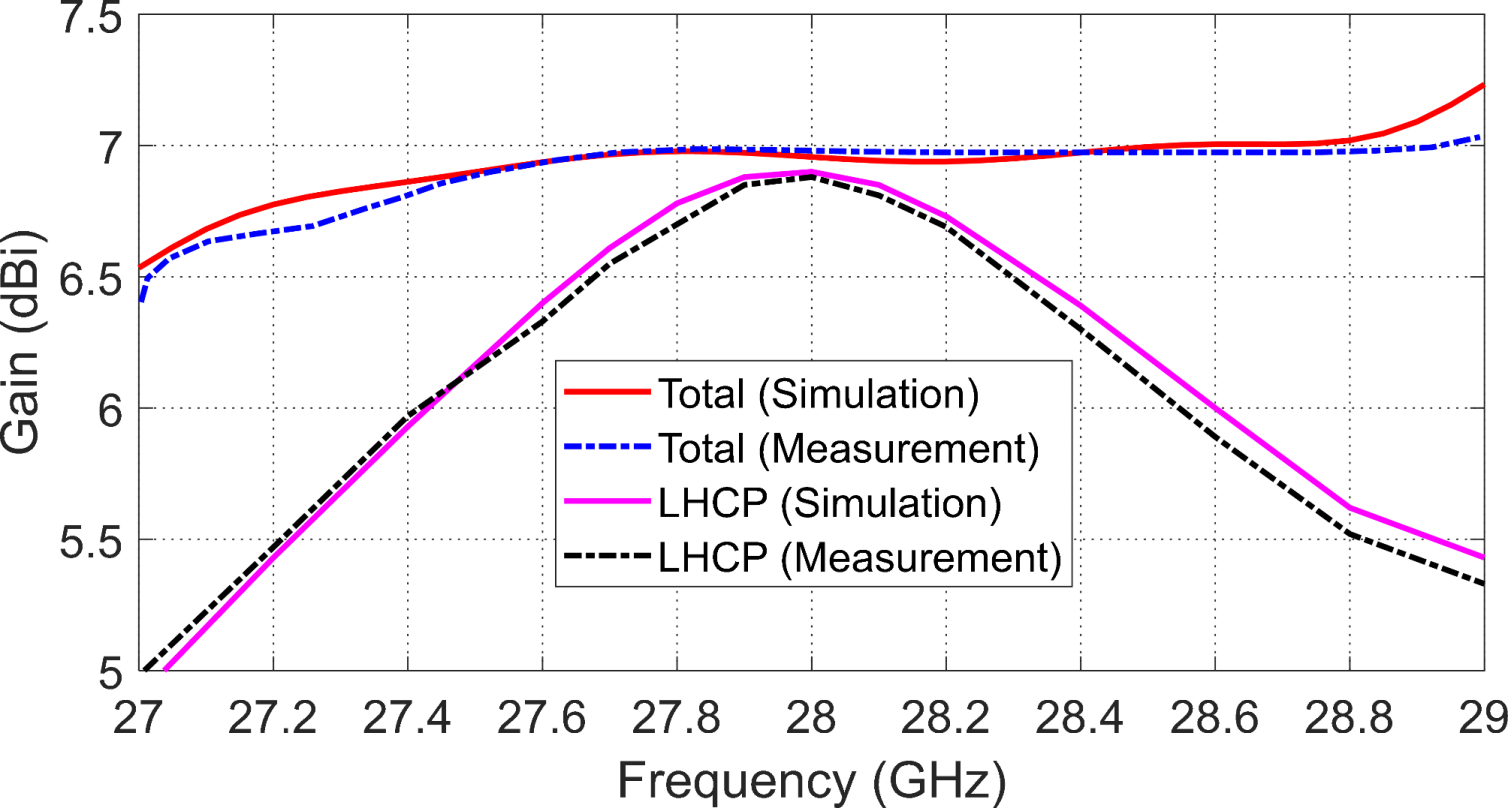
Variation of the maximum gain of the designed antenna with frequency.

The maximum gain variation of the designed antenna over the frequency range of $27-\:29\:\text{G}\text{H}\text{z}$ is illustrated in Fig. 21. It is shown that the maximum gain reaches a value of $7\:\text{d}\text{B}\text{i}$ at $28\:\text{G}\text{H}\text{z}$. The variation of the maximum gain value of the LHCP field with frequency is presented in Fig. 19. It is shown that the peak value is about $6.9\:\text{d}\text{B}\text{i}\text{c},$ and it occurs at $28\:\text{G}\text{H}\text{z}$. Thus, the gain of the circularly polarized field is close to the gain of the total radiated field over the frequency range of $27.8\:-\:28.2\:\text{G}\text{H}\text{z}$, which indicates perfect circular polarization. As shown in Fig. 21, the results of simulation are close to those of measurements. Both simulation and measurements show that the maximum gain of the total field is almost equal to $7\:\text{d}\text{B}\text{i}$ over the frequency range of $27.5-28.5\:\text{G}\text{H}\text{z}$, whereas the gain of the LHCP field ranges from $6.7$ to $6.9\:\text{d}\text{B}\text{i}\text{c}$ over the range of $27.8\:-\:28.2\:\text{G}\text{H}\text{z}$.

### Radiation Efficiency variation with frequency

**Fig. 22:**
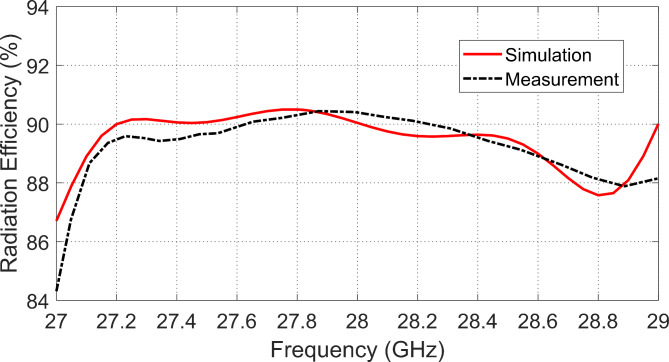
Variation of the radiation efficiency of the designed antenna with frequency.

The simulation results concerned with the variation of radiation efficiency versus frequency are presented in Fig. 22 in comparison to the measured response. Both results of simulation and measurements agree well with each other showing that the radiation efficiency of the designed antenna is kept above 90% over the whole frequency band of impedance matching ($27.5-28.5\:\text{G}\text{H}\text{z}$).

## Comparison with published work

The key innovations in the proposed antenna design can be summarized as follows. First, the patch geometry features 45°-diagonal symmetry, which achieves perfect circular polarization (0.1 dB axial ratio) at exactly 28 GHz, using a compact single patch. Additionally, simple defects in the ground structure are used to further enhance the circular polarization—an approach not previously reported in the literature. Another novel feature is the impedance matching achieved by tapering the feeding microstrip line. These innovations, combined with the compact size, perfect polarization, and excellent radiation pattern, make the antenna a strong candidate for MIMO systems and beamforming arrays.

The performance of the proposed CP antenna is compared to those of other CP antennas operating at 28 GHz and presented in some recently published work. The most important performance parameters constituting the subject of comparison are listed in Table 2. Since the primary focus of this work is to present a planar low-profile CP antenna as a building block for CP systems and arrays used in beamforming and beam steering, the comparison with the published works is limited to the design of single-element antennas. Therefore, the works listed in Table 2 for comparison specifically introduce CP single-element antennas with planar structures.

By limiting the design in this work to a compact single-element planar microstrip patch antenna, we expect the gain and bandwidth to be constrained. However, antennas with three-dimensional structures, such as those incorporating horns or dielectric lenses, can achieve circular polarization with higher gain (see^12^, for example). Also, antenna arrays of^13–16^ and MIMO antenna systems of^17,18^ can produce circular polarization with both high gain and wide frequency band at 28 GHz. However, the primary objective of the proposed design is not to achieve high gain, but rather to serve as a building block for CP antenna systems such as MIMO antennas, beam steering, and beamforming antenna arrays operating at 28 GHz.

The antenna proposed in this work exhibits low profile, perfect impedance matching, excellent circular polarization and high radiation efficiency. It is designed to produce LHCP, and it can be easily adapted to produce RHCP by simply mirroring its geometry along the feed line axis ($y$-axis). In this case, there is no need to repeat the simulations or experimental evaluations for the RHCP antenna, as it will yield identical results to those of the LHCP antenna, except for the sense of polarization in the far field.

**Table 2:** Comparison with CP antennas presented in recently published work and operating at $28\:\text{G}\text{H}\text{z}$ .

Work	Size $\text{m}\text{m}\times\:\text{m}\text{m}\times\:\text{m}\text{m}$		Min. $\left|{S}_{11}\right|\:\left(\text{d}\text{B}\right)$	Min. AR (dB)	Frequency Band (GHz) $\left|{S}_{11}\right|<-10\:\text{d}\text{B}$	Frequency Band (GHz) $\text{A}\text{R}<-3\:\text{d}\text{B}$	Sense of CP	Gain ($\text{d}\text{B}\text{i}\text{c}$)	Radiation Efficiency
[4]	$2\times\:2\times\:1.6$		$-18$	$1.2$	$26.5-28.7$	$26.5-28.7$	RHCP	$2.2$	$95\%$
[5]	$4.5\times\:7.9\times\:0.254$		$-22$	NA	$25.44-28.37$	$27.95-28.37$	NA	$7.48$	$97.7\%$
[6]	$3\times\:3\times\:0.508$		$-37$	$0.04$	$27-28.8$	NA	RHCP	$6.5$	NA
[7]	$3.3\times\:3.3\times\:0.25$		$-30$	$2.1$	$27.8-28.8$	$27.85-28.15$	RHCP	$7.3$	$91\%$
[Present]		$2.93\times\:2.93\times\:0.25$	$-35$	$0.5$	$27.5-28.5$	$27.8-28.2$	LHCP	$7.0$	$90\%$

## Conclusion

A compact CP patch antenna has been proposed for mm-wave applications within a frequency band around 28 GHz. The antenna is printed on a substrate with DGS. The geometry of the patch and the DGS are symmetric about a $45^\circ$-diagonal to produce perfect RHCP. A three-stage microstrip line that acts as an impedance transformer to match the antenna impedance to the $50\,{\Omega\:}$ source. The primary objective of the proposed design is not to produce a high-gain antenna, but rather to produce a low-profile planar antenna to serve as a building block for CP MIMO antenna systems, beam steering, and beamforming antenna arrays operating at 28 GHz. Both simulation and experimental results have shown that the antenna has $\left|{S}_{11}\right|<-35\:\text{d}\text{B}$ and $\text{A}\text{R}<0.5\:\text{d}\text{B}$ at $28\:\text{G}\text{H}\text{z}$. The surface current on the printed patch at the resonant frequency is presented to demonstrate the mechanism of producing circular polarization. The input impedance is matched to the feeding line over 1 GHz band, from 27.5 GHz to 28.5 GHz. The radiated wave is CP with $\text{A}\text{R}<3\:\text{d}\text{B}$ over about 0.4 GHz band starting from 27.8 GHz to 28.2 GHz. The peak gain of the LHCP field is $6.9\:\text{d}\text{B}\text{i}\text{c}$, and the radiation efficiency exceeds 90% all over the band of circular polarization.

## Data Availability

The datasets used and/or analysed during the current study are available from the corresponding authors on reasonable request.
